# An empirical study of integration models and mechanisms for maternity care in innovative vocational education

**DOI:** 10.1038/s41598-025-31951-w

**Published:** 2025-12-10

**Authors:** Weitai Luo

**Affiliations:** Guangxi Vocational and Technical College of Quality Engineering, Guangxi, China

**Keywords:** Vocational education, Simulation-based training, Patient satisfaction, Student–patient interaction, Perceived maternal complications, Health care, Medical research

## Abstract

Integrated vocational education is increasingly essential for addressing maternal health disparities in under-resourced regions of China. Persistent workforce shortages and fragmented training models have contributed to inconsistent care delivery, particularly in Western provinces. This study evaluated an integrated model that combines simulation-based learning, structured supervision, and early clinical immersion to enhance training effectiveness. A convergent parallel mixed-methods design assessed the model’s impact on patient satisfaction, student interaction, and perceived maternal complications. Quantitative data were collected from 300 participants, including students, maternity nurses, clinical instructors, and postnatal patients, and analyzed using SPSS and partial least squares structural equation modeling (SmartPLS). Qualitative insights from 14 semi-structured interviews were analyzed thematically using NVivo. The integrated training model significantly predicted higher patient satisfaction (β = 0.863, R² = 0.743), aligning with Kolb’s Experiential Learning Theory. However, no significant relationships were found between student interaction, patient satisfaction, or perceived maternal complications. Critically, the staff training construct demonstrated weak psychometric validity (CR = 0.337; AVE = 0.126), warranting cautious interpretation of its statistical strength. Qualitative findings provided essential context, revealing fragmented implementation, unclear student roles, and institutional barriers such as emergency delays and staffing shortages. These systemic constraints help explain the absence of improvement in clinical outcomes despite enhanced satisfaction ratings. In conclusion, integrated vocational education can enhance perceived care quality, but its clinical impact depends on institutional readiness and supervisory fidelity. This study offers actionable insights for policy reforms under the Healthy China 2030 agenda and supports scalable models for maternal health training in low-resource settings.

**Supplementary Information:**

The online version contains supplementary material available at 10.1038/s41598-025-31951-w.

## Introduction

### Background and rationale

China has made significant strides in reducing maternal mortality, with the national maternal mortality ratio declining to 16.1 per 100,000 live births as of 2020^1^. Maternal health reforms in China have produced substantial gains in mortality reduction, yet progress remains uneven, especially across western provinces such as Gansu, Guizhou, and Yunnan, where maternal mortality ratios and obstetric complication rates remain above national averages^2^. These regional disparities are compounded by workforce shortages, limited supervisory capacity, and an underdeveloped vocational training infrastructure. National policy frameworks, including the Healthy China 2030 initiative, have prioritized maternal health and professional training as key levers for improving care quality^3^. Despite these commitments, China’s vocational nursing and midwifery programs continue to rely heavily on theoretical instruction, often delaying or diluting clinical exposure. This disconnect between academic learning and frontline practice has been linked to gaps in emergency preparedness, communication skills, and maternal outcomes^4,5^. While simulation-based training and early clinical immersion have been introduced in selected pilot sites, their use remains fragmented and poorly evaluated. For example, Sun et al.^6^ documented improvements in student confidence and skill acquisition following simulation in one provincial program, but no national impact assessments have been conducted. In contrast, countries such as the United Kingdom, Finland, and Australia have institutionalized simulation-based vocational training in maternal care, demonstrating measurable reductions in obstetric errors and improved team-based care (WHO, 2021). However, the evidence base in China is limited by overreliance on cross-sectional studies, small samples, and an absence of multi-level implementation research. To address these gaps, this study employs a convergent parallel mixed-methods design to evaluate an integrated vocational training model, examining its effects on patient satisfaction, student engagement, and maternal outcomes across public institutions in western China.

While policy reforms in China have emphasized competency-based learning and maternal health workforce expansion, multiple structural and empirical gaps persist in vocational education for maternity care. First, most programs still lack systematic integration of simulation-based training and structured clinical supervision, key components shown to improve emergency response and clinical judgment in international settings^7^. Where simulation is present, its implementation remains fragmented, lacking national evaluation mechanisms or fidelity metrics^8^. Second, the existing literature tends to prioritize student performance indicators such as examination scores or self-reported satisfaction over patient-centered outcomes like complication reduction or care quality. Few studies have examined the mediating role of institutional supervision, student-patient interaction, or training environment on maternal health outcomes. Third, research on implementation fidelity and contextual barriers such as workload, role ambiguity, and resource constraints is notably limited in China’s vocational education literature. Moreover, studies often rely on cross-sectional survey data or single-institution case studies, limiting generalizability and the ability to draw causal inferences. There is a lack of rigorous, mixed-methods evaluations that examine both quantitative impacts and qualitative mechanisms of integrated training interventions. This study addresses those gaps by applying a convergent parallel mixed-methods design to evaluate how simulation, supervision, and student engagement influence patient satisfaction, perceived maternal complications, and institutional readiness in public maternity care institutions across under-resourced Chinese provinces^9^.

Building on identified gaps in vocational maternity care training, this study aims to evaluate the impact, fidelity, and contextual effectiveness of an integrated vocational training model incorporating simulation-based instruction, structured clinical supervision, and early-stage clinical immersion in public maternity care institutions across western China. The specific objectives are to: (a) Quantitatively assess the effect of integrated vocational training on patient satisfaction, student–patient interaction, and reported maternal complications, (b) Evaluate the psychometric reliability and structural validity of vocational training constructs using PLS-SEM, (c) Qualitatively explore the experiences of students, nurses, instructors, and patients regarding training quality, supervisory practices, and implementation barriers, and (d) Integrate quantitative and qualitative findings to assess implementation fidelity and institutional readiness for reform across low-resource settings.

This study offers both theoretical and practical contributions to the field of maternal vocational education. It represents one of the few mixed-methods, role-stratified evaluations in the Chinese context that links training integration via simulation, supervision, and early clinical immersion to both patient-centered outcomes and institutional implementation. By employing SPSS, SmartPLS for structural modeling, and NVivo for qualitative triangulation, the study delivers a multi-level, empirically grounded assessment of reform impact^10^.

## Literature review

Vocational education in maternal and neonatal health has undergone substantial reform globally over the past two decades, driven by rising expectations for workforce readiness in high-risk obstetric environments^11^. Traditionally, vocational curricula in low and middle-income countries (LMICs) emphasized didactic instruction and delayed clinical exposure, resulting in gaps in skill transfer, emergency response, and professional identity development^12^. In contrast, high-income countries, including the UK, Australia, and Finland, have implemented integrated models that blend simulation, structured supervision, and early clinical immersion. These models have been associated with improved patient safety metrics, enhanced student confidence, and reduced obstetric errors^13^. In China, the “Healthy China 2030” strategy identifies maternal care quality and workforce training as national priorities, emphasizing the need for competency-based midwifery and nursing education. Several provincial pilot programs now incorporate simulation-based obstetric scenarios and clinical mentorship, particularly in higher-tier urban institutions. However, implementation remains uneven across vocational colleges, particularly in under-resourced western provinces, where supervision structures are weak, simulation tools are underutilized, and faculty capacity is limited. These disparities reflect broader institutional readiness challenges that constrain the translation of reform ambitions into clinical outcomes, especially in maternal complications and continuity of care. To date, few rigorous evaluations have assessed how these evolving vocational models influence both student learning and maternal health outcomes.

Globally, integrated vocational education in maternal care is anchored on three interdependent pillars: (1) simulation-based learning, (2) structured supervision, and (3) early-stage clinical immersion^14^. Simulation enables students to practice rare but critical emergencies such as postpartum hemorrhage or shoulder dystocia within low-risk, high-realism environments^15^. Meta-analyses confirm that high-fidelity simulation improves technical proficiency, decision-making, and team-based communication in obstetric care^16^. Supervision quality is equally essential. Evidence from both high-income settings and LMICs, including Uganda and Nepal, shows that poorly supervised clinical exposure reduces student engagement to passive observation, impeding skill acquisition and delaying professional identity formation. Structured supervision characterized by clear role expectations, formative feedback, and sustained instructor presence has been associated with greater learner autonomy and confidence^17,18^. These findings align with Bandura’s Social Learning Theory and Eraut’s Informal Learning Theory, which emphasize modeled behavior and real-time social interaction as catalysts for workplace learning. By contrast, structured supervision involving feedback loops and defined student roles enhances performance and professional identity formation. In China, the implementation of clinical mentoring in vocational nursing programs has shown promise but remains under-evaluated in maternity-specific domains. Early-stage clinical immersion further accelerates competence when integrated with simulation and supervision. However, the literature also cautions against “pseudo-immersion,” wherein students are physically present in clinical spaces but remain functionally disengaged due to institutional disconnects between education providers and clinical sites^4,19^. This pattern is particularly evident in LMICs and under-resourced regions of China, where staffing shortages, unclear student roles, and simulation underutilization compromise implementation fidelity. Despite China’s policy-level efforts to embed these components into vocational reform, few institutions possess the infrastructure, faculty capacity, and quality assurance mechanisms necessary for high-fidelity execution.

Despite the growing emphasis on integrating simulation, supervision, and clinical immersion into vocational maternity training, evaluations of these models remain limited in both scope and analytical depth. Most existing studies focus narrowly on student outcomes, typically measured via self-reported satisfaction or post-training knowledge assessments, without incorporating the perspectives of other key stakeholders, such as clinical instructors, patients, or maternity ward nurses^4,20^. As a result, the broader institutional and interpersonal dynamics that shape vocational training impact remain underexplored. Furthermore, outcome-oriented evaluation is rare. While simulation training is often credited with improving student confidence or technical skills, few studies link these gains to clinical outcomes such as maternal complication rates, patient satisfaction, or care continuity. This gap is particularly pronounced in China, where reforms under Healthy China 2030 have promoted training integration, but evaluations remain anchored in curricular or structural indicators, not patient-facing results.

Another overlooked dimension is implementation fidelity. Even where integrated models exist, few studies assess whether simulation-based instruction, supervision protocols, and early immersion were implemented consistently or at scale. Without fidelity metrics, it is difficult to attribute observed outcomes to the training intervention itself rather than contextual variability. Moreover, the absence of triangulated, role-stratified data obscures how different participants interpret and experience the same program^21^. This study addresses these limitations by employing a mixed-methods, role-stratified design that examines how simulation, supervision, and student engagement affect outcomes across patients, students, and providers, while accounting for fidelity and institutional context.

In response to these persistent gaps in implementation fidelity, outcome evaluation, and stakeholder stratification, researchers increasingly call for theory-driven, mixed-methods evaluations in vocational health education. Theoretical frameworks offer the scaffolding necessary to move beyond descriptive metrics and toward explanatory insight. Kolb’s Experiential Learning Theory underscores the role of active, iterative practice in skill acquisition, while Bandura’s Social Learning Theory emphasizes the importance of modeled behavior and feedback within supervised environments. Raut’s Informal Learning model accounts for the significance of unstructured, real-time clinical exposure, and Weiner’s Organizational Readiness Theory provides a lens to evaluate whether institutions possess the structural and psychological conditions for change^22^. China’s vocational health education system now stands at a pivotal moment. National policy ambitions, articulated in the Healthy China 2030 agenda, seek to align maternal care training with global standards of quality and patient safety. However, this aspiration requires empirical models that not only measure training inputs but also examine implementation mechanisms and real-world outcomes. As global evidence increasingly confirms, the effectiveness of integrated training depends not solely on curriculum content but on the fidelity of execution, clarity of learner roles, and responsiveness to institutional context^23^. This study responds to these research directions by integrating structural equation modeling with thematic qualitative analysis to examine how integrated training models influence maternal care outcomes across roles and contexts in public institutions in western China.

## Methodology

### Study design

This study followed a convergent parallel mixed-methods design as described by Creswell and Plano (2018), in which quantitative and qualitative strands are collected and analyzed independently and then integrated at interpretation. The quantitative strand tested hypothesized relationships using partial least squares structural equation modeling (PLS-SEM), while the qualitative strand explored participant experiences and implementation barriers through thematic analysis. Data were collected independently, analyzed concurrently, and integrated at the interpretation stage to ensure triangulated insight^24^. A quasi-experimental, post-intervention design was applied. The experimental group received an integrated training package comprising simulation-based learning, structured clinical supervision, blended e-learning, and early clinical immersion. The control group followed standard institutional curricula without these enhancements. This mixed-methods approach was selected to capture both measurable outcomes and contextual mechanisms, allowing a more comprehensive evaluation of training effectiveness and implementation fidelity across real-world vocational and clinical settings^25^.

The integrated training package was provided in 6 weeks and included: (1) high-fidelity simulation training (3 sessions in total, 2 h each) on the topic of common obstetric emergencies (postpartum hemorrhage, shoulder dystocia, neonatal resuscitation), (2) structured clinical supervision during a 3-week period of early clinical immersion in the maternity ward with daily supervisory check-ins, and (3) integrated e-learning modules (four brief modules, approximately 4 h in total) on the topics of communication and patient The experienced clinical instructors of the affiliated vocational college provided simulation and supervision, and senior maternity nurses at each site. The fidelity was measured using a checklist that was filled by the supervisors at the end of every simulation and by spot-checks of the lead author (attendance logs and supervisor checklists can be obtained on demand).

### Participants and sampling strategy

A total of 300 participants were recruited through purposive, role-stratified sampling across three public maternity care institutions and affiliated vocational colleges in western China. The quantitative strand included 120 nursing students (60 experimental, 60 control), 90 postnatal patients, 45 maternity nurses, and 45 clinical instructors. Assignment to experimental and control groups was determined by the institutional training schedule, consistent with quasi-experimental, post-intervention designs. Although longitudinal data collection was planned, baseline measures were not feasible due to delays in program rollout^26^. Sample size adequacy was ensured through both G*Power 3.1 analysis (minimum *N* = 138 for medium effect sizes at 95% power) and the “10-times rule” for PLS-SEM structural paths, justifying the total *N* = 300 for multigroup and subgroup analysis^27^. For the qualitative strand, 14 participants were purposively selected from across stakeholder groups (4 students, 4 postnatal patients, 3 maternity nurses, 3 clinical instructors). Selection criteria included direct involvement in or exposure to the integrated training model. Data saturation was reached by the 13th interview^28^. All interviews were conducted in Mandarin, transcribed verbatim, translated into English by certified professionals, and verified through back-translation for conceptual and linguistic fidelity^29^.

### Quantitative instruments and measures

The quantitative instrument consisted of four constructs: Staff Training (9 items), Student Interaction (8 items), Patient Satisfaction (7 items), and Perceived Maternal Complications (7 items). Each construct was developed based on relevant literature and mapped to the study’s conceptual framework, incorporating dimensions of simulation, supervision, communication, and care outcomes. Items were rated using a 7-point Likert scale, ranging from 1 (Strongly Disagree) to 7 (Strongly Agree), allowing for greater response sensitivity across roles^30^. The Staff Training scale measured instructional quality, simulation practices, and supervisory support. Student Interaction assessed engagement and role clarity during clinical placement. Patient Satisfaction focused on communication, respect, and care responsiveness. Perceived Maternal Complications captured patients’ self-reported experiences with delays, safety concerns, or postnatal difficulties. Items were reviewed by clinical educators and piloted for clarity before full-scale administration. All constructs were tested for internal consistency and convergent validity using SPSS v26 and SmartPLS 4. Reliability was assessed via Cronbach’s alpha, while construct validity was confirmed through composite reliability (CR), average variance extracted (AVE), and discriminant validity (HTMT ratio) by current structural equation modeling standards^31^. The full list of scale items is provided in Supplementary Table S1.

### Qualitative data collection

The sample of those who took part in the in-depth interviews was purposely chosen to reach role stratification and a variety of views (4 students, 4 patients, 3 maternity nurses, 3 clinical instructors). Qualitative interview inclusion criteria were direct participation in or exposure to the integrated training model within the period of intervention and willingness to participate in the interview. All participants were excluded because of their inability to give informed consent or low recall of the training period. Saturation of themes and maximum variation were the reasons to interview 14 participants; the 12th transcript reached the saturation, and the last two interviews supported the themes. This methodology is based on the conventional mixed-methods recruitment practices, that purposive sampling to gain in-depth qualitative insights and pragmatic recruitment to allow generalization of the findings.

To understand the experiences of the participants using the integrated maternity-care training model, semi-structured qualitative interviews were held with participants who were selected purposively. The interviews were conducted in a separate room in each of the study locations by trained interviewers who were not part of the teaching or the assessment. The interviews were recorded on audio with permission and took about 25–40 min.

In order to be transparent and consistent, the interviews were structured to be guided by a set of open-ended questions. The core questions were such as:

1. Please provide details of the training you received during your period of study and its impact on your bedside role.
2. Please explain a particular clinical case in which the training was beneficial or not?
3. What were the supervisors like when you were being trained, and what kind of feedback did you get?
4. In your view, were students involved in patient care? If so, in what ways?
5. What were the obstacles that you saw impeding the impact of the training on clinical outcomes?

The entire semi-structured interview guide is attached in Supplementary File S2.

Though it was planned to collect data in three phases (baseline, midterm, post-intervention) data collection model, the baseline data could not be collected because of the delayed rollout of the program and institutional schedule limitations across the three sites that participated. The cohorts of training were initiated at various times and access to the wards during the proposed baseline period was limited because of institutional staffing rotations and patient-flow policies. This led to the study taking the form of a quasi-experimental post-test only design where all outcome measure was undertaken right after the training period.

### Data collection procedures

Data collection followed a convergent parallel mixed-methods design, with quantitative and qualitative data gathered concurrently. Although a three-phase (baseline, midterm, post-intervention) model was initially proposed, only post-intervention data were collected due to rollout constraints, aligning the study with a quasi-experimental post–test–only design^32^. Structured questionnaires were administered to all participant groups following program completion. Data were collected in person by trained enumerators who followed a standardized protocol. Assignment to experimental or control groups was verified through institutional records. For the qualitative strand, semi-structured interviews were conducted using a guide based on the Knowledge, Attitudes, and Practices (KAP) framework. Fourteen participants (students, patients, nurses, and instructors) were purposively selected, and data saturation was confirmed by the 13th interview^33^. All interviews were conducted in Mandarin, audio-recorded with consent, transcribed verbatim, and professionally translated into English. To ensure conceptual accuracy and linguistic equivalence, translations were cross-checked using back-translation and bilingual review procedures, maintaining the integrity of cross-language qualitative analysis.

### Data analysis

Initial statistical checks and reliability assessments were conducted using SPSS v26. All four constructs demonstrated strong internal consistency, with Cronbach’s alpha values exceeding 0.90. Sampling adequacy was verified via a Kaiser−Meyer−Olkin (KMO) value of 0.943, and Bartlett’s Test of Sphericity was significant (*p* <.001), confirming the dataset’s suitability for factor analysis^34^. SmartPLS 4.0.9 was used to perform structural equation modeling (PLS-SEM), which is suitable for small-to-moderate sample sizes and complex models involving both reflective and formative constructs. Measurement model validity was evaluated using outer loadings, Average Variance Extracted (AVE), Composite Reliability (CR), and Heterotrait-Monotrait (HTMT) ratios. Structural model analysis included path coefficients (β), t-values, R², Q², and Standardized Root Mean Square Residual (SRMR)^35,36^. Significance testing was conducted via bootstrapping with 5,000 subsamples, and multi-group analysis (MGA) was used to compare control and experimental groups^37^. All analytical procedures followed established guidelines for PLS-SEM^38^, ensuring methodological rigor and replicability^39^.

Thematic analysis was conducted in NVivo 14 following Braun and Clarke’s six-phase framework^40^. Transcripts were independently coded by two researchers to ensure analytic credibility. Disagreements in initial coding were resolved through consensus meetings, and final inter-coder reliability reached Cohen’s Kappa = 0.81, indicating high agreement^41^. An audit trail was maintained to document coding decisions, analytic memos, and theme development across iterations. Codes were developed inductively and clustered into four overarching themes and ten subthemes, aligned with the study’s conceptual constructs: Staff Training, Student Interaction, Patient Satisfaction, and Perceived Maternal Complications. NVivo tools, including matrix queries and clustering, were used for conceptual triangulation^42^. Thematic saturation was reached by the twelfth interview, with the thirteenth and fourteenth transcripts reinforcing the themes. Quotes were selected to reflect diverse roles and themes. Results were later integrated with the SEM model to support or explain statistical patterns across constructs and paths.

### Ethical considerations

Ethical approval was obtained from the institutional review boards of the participating universities and clinical sites^43^. Written informed consent was secured from all participants after a full explanation of the aims and procedures^44^. Confidentiality was maintained using coded identifiers, and data were encrypted and securely stored. Institutional permissions were granted by training institutes and maternity wards. Participants retained the right to withdraw at any stage, and debriefing was conducted after interviews^45^. A qualitative ethics log and audit trail were archived to ensure transparency and compliance with international research standards.

## Results

### Quantitative results

This section presents quantitative findings assessing the relationships between vocational staff training, student interaction, patient satisfaction, and perceived maternal complications. Partial Least Squares Structural Equation Modeling (PLS-SEM) was employed, given its appropriateness for small-to-moderate sample sizes and models with both reflective and formative constructs^38^. Data from 300 participants (see Table 1) were analyzed using SmartPLS 4.

**Table 1:** Participant demographic characteristics (*N* = 300).

Participant Role	Group	*n* (%)	Mean Age (SD)	Gender (% Female)	Additional Notes
Students	Experimental	60 (20%)	22.4 (1.8)	100%	Received simulation-based, supervised training
Students	Control	60 (20%)	22.6 (1.5)	100%	Received conventional vocational training
Postnatal Patients	Not Applicable	90 (30%)	27.3 (3.2)	100%	Recruited from public maternity wards
Clinical Instructors	Not Applicable	45 (15%)	38.9 (4.5)	73%	Involved in student supervision
Maternity Nurses	Not Applicable	45 (15%)	35.2 (5.1)	80%	Provided care during the observational period
Total		300	–	–	

Descriptive breakdown of participants by role (students, patients, nurses, instructors), group allocation (experimental/control), gender, and mean age.

All constructs demonstrated strong internal consistency (Cronbach’s α > 0.90): Staff Training (0.905), Patient Satisfaction (0.932), Perceived Maternal Complications (0.931), and Student Interaction (0.900). However, the Staff Training construct failed to meet convergent validity thresholds. While item ST9 showed strong loading (0.987), the remaining indicators performed poorly (e.g., ST6 = 0.054), yielding critically low AVE = 0.126 and CR = 0.337. In contrast, the remaining constructs met acceptable validity criteria (AVE > 0.50, CR > 0.85, HTMT < 0.85)^38^.

**Fig. 1:**
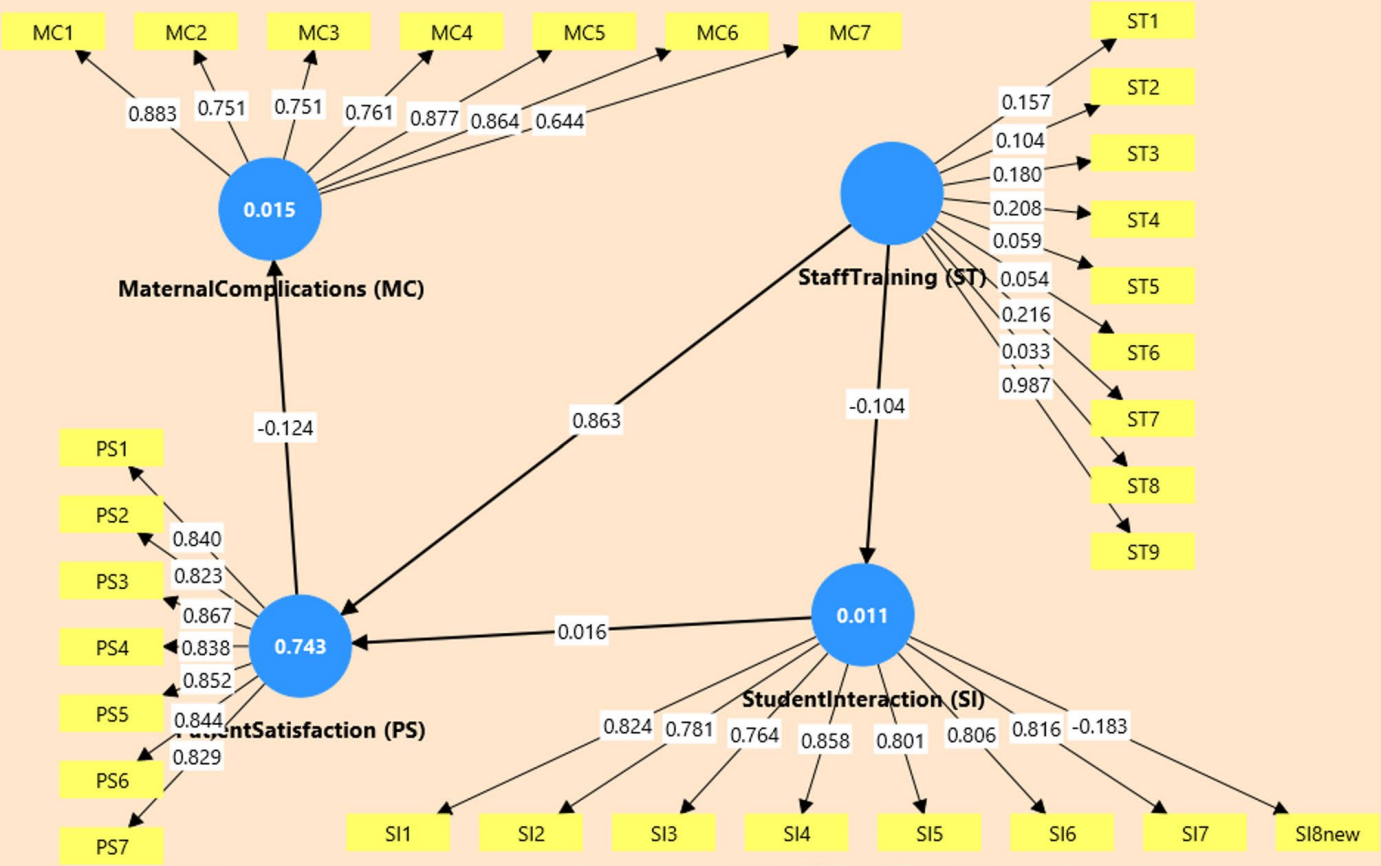
PLS-SEM Structural Model.

Despite this measurement limitation, the structural model revealed a significant path from Staff Training to Patient Satisfaction (β = 0.863, t = 8.123, *p* <.001), as shown in Fig. 1. Other hypothesized paths were not statistically significant: ST → SI (β = − 0.104, *p* =.470), SI → PS (β = 0.016, *p* =.720), and PS → MC (β = − 0.124, *p* =.399), indicating limited influence of training on student engagement or clinical complications. Model explanatory power was high for Patient Satisfaction (R² = 0.743), but negligible for Student Interaction (0.011) and Perceived Maternal Complications (0.015) (see Table 2). Predictive relevance (Q²) followed a similar pattern: high for PS (0.717), negative for SI (–0.006), and MC (–0.004). The SRMR value = 0.168, above the recommended threshold (0.08), suggests limited global fit. However, in exploratory PLS-SEM, predictive strength (R², Q²) is prioritized over overall model fit^35^.

**Table 2:** Structural model output: path Coefficients, R², and Q² Values.

Construct	Cronbach’s α	AVE	CR	*R*²	Q²	Significant Paths ((β)
Staff Training (ST)	0.905	0.126	0.337	–	–	ST → PS (0.863***), ST → SI (–0.104)
Patient Satisfaction (PS)	0.932	0.709	0.945	0.743	0.717	PS → MC (–0.124)
Maternal Complications (MC)	0.931	0.631	0.922	0.015	0.004	–
Student Interaction (SI)	0.900	0.575	0.898	0.011	0.006	SI → PS (0.016)

PLS-SEM results for hypothesized paths across constructs, including explanatory and predictive power for each endogenous variable. *p* <.001, all other paths non-significant (ns), CR = Composite Reliability, AVE = Average Variance Extracted.

The strong ST → PS effect supports Kolb’s Experiential Learning and Bandura’s Social Learning Theory, underscoring the importance of supervised experiential learning for perceived care quality^46^. The lack of significant effects on Student Interaction and Perceived Maternal Complications likely reflects low-fidelity, lecture-based training environments. These findings are further explained by Raut’s Informal Workplace Learning Theory, which posits that critical professional knowledge is often acquired through unsupervised and poorly supported real-world exposure^47^.

In summary, while integrated vocational training appears effective in enhancing patient satisfaction, it shows limited impact on student engagement or complication reduction. These results emphasize the need for better-integrated, high-fidelity simulation and supervision models.

### Qualitative results

Interviews and focus group discussions with 14 participants, including nursing students, clinical instructors, and postnatal patients, were thematically analyzed using NVivo 14^48^. Codes were developed inductively and grouped into four main themes: inconsistent training delivery, passive student roles, perceived care quality, and system-level barriers. These are clustered under two domains: training process challenges and outcome-related perceptions. Participants described a wide variation in instructional methods, with some exposed to simulation-based learning and others only observing. As one student explained, “We just stood back and watched; we never practiced ourselves.” Staff echoed this, noting inconsistent supervision structures and limited time for hands-on mentoring. Patients rarely acknowledged student involvement, aligning with the low explanatory power of Student Interaction in the SEM model. In contrast, staff confidence and communication strongly shaped perceived care quality. A patient shared, “The nurse who smiled and explained things made me feel like I wasn’t alone.” Yet even in positive experiences, maternal outcomes were seen as constrained by resource shortages and delays. These insights help explain the non-significant Patient Satisfaction → Perceived Maternal Complications path in the quantitative model. Thematic saturation was achieved, and credibility was enhanced through peer debriefing^49^. Overall, the qualitative findings align with Raut’s Informal Learning Theory, illustrating how informal, unsupported clinical experiences often undermine formal training intentions^50^. Quotes were selected to represent a balance across all participant types.

### Group comparison results

A post-intervention group comparison was conducted to assess the impact of the integrated vocational training model^51^. Of the total 120 students, 60 students were enrolled in the experimental group, which followed a structured training program incorporating simulation, blended learning, and early-stage clinical exposure^52^. The remaining 60 students formed the control group, engaged in a traditional curriculum characterized by theoretical instruction delivered separately from delayed clinical exposure^53^. The group assignment was based on institutional enrollment rather than randomization. While baseline data were planned per the proposal’s longitudinal design, real-world delays in program rollout prevented pre-intervention data collection^54^.

The experimental group demonstrated superior measurement model performance, with high internal consistency and convergent validity for constructs such as Staff Training and Patient Satisfaction (CR > 0.90; AVE > 0.60)^55^. Item ST9, reflecting perceived clinical preparedness, showed an exceptionally high loading (0.987). In contrast, the control group exhibited inconsistent loadings across Staff Training items, including ST6 (0.054), resulting in weaker construct reliability and lower construct clarity.

Bootstrapped Multi-Group Analysis (MGA) using PLS-SEM revealed that the Staff Training → Patient Satisfaction path was significantly stronger in the experimental group (β = 0.863, t = 8.123, *p* <.001) compared to the control group, where the effect was weaker and non-significant. No significant group differences were observed for Student Interaction → Patient Satisfaction or Patient Satisfaction → Perceived Maternal Complications, indicating that student engagement and downstream clinical outcomes remain constrained under both training conditions.

The model’s explanatory power was notably higher in the experimental group, accounting for 74.3% of the variance in Patient Satisfaction (R² = 0.743) and demonstrating strong predictive relevance (Q² = 0.717). By contrast, the control group model demonstrated substantially lower predictive and explanatory power (R² < 0.40; Q² ≈ 0). Both models explained minimal variance for Student Interaction and Perceived Maternal Complications (R² < 0.02), suggesting persistent institutional barriers to improving student engagement and reducing clinical risk through training alone (see Table 2 for group-wise path estimates and construct metrics).

These findings affirm the effectiveness of the integrated training model in improving perceived care quality while also revealing its current limitations. Although the intervention significantly enhances satisfaction, it does not yet translate into improved student participation or maternal outcomes, reflecting gaps in supervision, engagement protocols, and emergency preparedness. These insights are consistent with Raut’s Informal Learning Theory, which emphasizes the importance of structured, guided workplace learning in shaping professional competence. Overall, while the integrated approach strengthens training perceptions and patient satisfaction, broader systemic reforms remain essential to maximize its impact on clinical safety and student development.

### Triangulation and integration

Triangulation of quantitative and qualitative findings revealed both alignment and divergence across constructs, offering a richer interpretation of the results^56^. As summarized in Table 3, Staff Training significantly predicted Patient Satisfaction, a finding reinforced by themes of relational trust and staff communication drawn from students, instructors, and patients. However, the training construct’s poor reliability was mirrored in narratives of fragmented implementation, inconsistent simulation use, and variable supervision, particularly among students in the control group^4^. This illustrates that statistical strength can coexist with conceptual weakness when implementation fidelity is low^57^.

**Table 3:** Triangulated summary of SEM constructs and NVivo themes:.

Construct (SEM)	Key SEM Finding	Qualitative Themes & Participant Roles (Group)	Partial (✓/✗)	Interpretive Commentary
Staff Training (ST)	Significantly predicts Patient Satisfaction (β = 0.863); low reliability (CR = 0.337, AVE = 0.126)	Inconsistent Training Delivery (Students – Control); Fragmented Integration (Students – Experimental)	✓	Although statistically strong, qualitative accounts reveal poor implementation fidelity, explaining weak internal consistency.
Student Interaction (SI)	Not predicted by ST (β = − 0.104), nor predictive of PS (β = 0.016); low R² = 0.011	Limited Engagement, Role Ambiguity (Students – Both); Patient Invisibility (Patients – Both)	✗	Students are unclear of roles, lack supervision, and are largely unrecognized, explaining weak SEM paths and aligning with informal learning theory.
Patient Satisfaction (PS)	Strongly predicted by ST (R² = 0.743); does not predict MC (β = − 0.124)	Relational Trust, Staff Communication (Patients – Both, Nurses – Both)	✓/✗	Satisfaction improved via staff communication, but this perception didn’t translate into reduced complications, suggesting a gap between affective and clinical outcomes.
Perceived Maternal Complications (MC)	Low explained variance (R² = 0.015); PS → MC path non-significant	Emergency Delays, Resource Scarcity, Staff Overload (Instructors, Nurses – Both)	✗	Maternal outcomes are governed more by structural limitations than satisfaction or training, supporting Organizational Readiness Theory.

In contrast, Student Interaction showed no significant relationships in the SEM model. This was reflected in qualitative accounts describing students as passive, underutilized, and often invisible in clinical settings. Participants described unclear expectations and weak supervisory protocols, suggesting that institutional culture, not curriculum design alone, shaped student engagement^58^. These insights support Raut’s Informal Learning Theory and align with Kolb’s model, in which meaningful learning is grounded in active participation. Although Patient Satisfaction was strongly explained by Staff Training (R² = 0.743), it failed to predict Perceived Maternal Complications. This disconnect was clarified through patient narratives, which emphasized interpersonal care but also revealed systemic barriers like emergency delays and staff shortages that satisfaction alone could not overcome. Staff and instructors echoed this concern, noting that maternal outcomes were more sensitive to institutional readiness than individual competence. These patterns underscore the applicability of Organizational Readiness Theory in explaining the null quantitative paths. In sum, while vocational training improves perceived care quality, its fragmented execution and unclear student roles undermine its broader impact. This integrated understanding highlights the need for stronger alignment between training models and institutional capacity. Without both, intended improvements in clinical outcomes may remain statistically insignificant even when perceptions of care are high. These triangulated insights reinforce the value of concurrent, mixed-methods evaluation in capturing both outcome patterns and the contextual mechanisms behind them.

## Discussion

This study evaluated the effects of an integrated vocational training model on student engagement, patient satisfaction, and maternal health outcomes through a quasi-experimental, convergent parallel mixed-methods design. Participants were divided into control and experimental groups, with the latter receiving simulation-based training, early clinical immersion, and structured supervision. Quantitative data from 300 respondents were analyzed using SPSS and SmartPLS, while qualitative data from 14 translated interviews spanning students, instructors, nurses, and patients were thematically analyzed using NVivo 14.

Quantitative results demonstrated a significant positive effect of staff training on patient satisfaction (β = 0.863, t = 8.123, *p* <.001), particularly within the experimental group (see Fig. 1; Table 1). However, no statistically significant effects were observed between staff training and student interaction, nor between student interaction and patient satisfaction. Likewise, patient satisfaction did not significantly reduce perceived maternal complications. Although mid-phase feedback indicated enhanced student confidence, these improvements did not translate into meaningful structural effects within the final model.

Qualitative insights contextualized the numerical findings. NVivo analysis identified four dominant themes: (1) Training Quality and Simulation Realism, (2) Student Role Clarity and Supervision, (3) Staff-Patient Communication and Emotional Safety, and (4) System-Level Barriers to Emergency Response (Table 4). Experimental group students consistently reported more meaningful clinical immersion, while patients emphasized the emotional impact of confident, communicative care providers. However, structural issues such as inadequate supervision and emergency preparedness persisted in both groups, offering explanations for the non-significant pathways to maternal outcomes.

**Table 4:** NVivo-Themed summary: Codes, Quotes, Constructs, and theoretical Anchors.

Theme/Domain	NVivo Code/Subtheme	Illustrative Quote	Participant Role (Group)	Mentions (*n*)	Linked Construct(s)	SEM Insight/Interpretation	Theoretical Anchor
Training Process Challenges	Lack of practical exposure	“We just stood back and watched; we never practiced ourselves.”	Student (Control)	11	Staff Training	Explains poor reliability of ST construct; most items loaded weakly except ST9	Raut’s Informal Workplace Learning
Training Process Challenges	Uneven use of simulation tools	“In our traditional batch, we didn’t use the mannequins, just watched videos.”	Student (Control)	9	Staff Training	Highlights fragmented delivery across training groups; supports ST group variance	Rraut’s Informal Workplace Learning
Training Process Challenges	Fragmented integration model implementation	“They said it’s integrated, but it felt like two separate courses.”	Student (Experimental)	8	Staff Training/Student Interaction	Shows mismatch between program design and actual delivery; weakens ST→SI path	Situated Learning Theory
Student Role Ambiguity	Limited clinical engagement	“Mostly, I just handed things over. Nobody asked us to assist.”	Student (Both)	9	Student Interaction	Explains low R² for SI; students are not functionally engaged	Raut’s Informal Workplace Learning
Student Role Ambiguity	Unclear expectations	“We’re never sure if we’re allowed to touch the patient or just observe.”	Student (Both)	7	Student Interaction	Reinforces structural weakness in SI measurement; lack of clarity limits effectiveness	Experiential Learning Theory (Kolb)
Perceived Care Quality	Staff communication	“The nurse who smiled and explained things made me feel like I wasn’t alone.”	Patient (Both)	7	Patient Satisfaction	Supports strong ST → PS path; perceived confidence improves satisfaction	Bandura’s Social Learning Theory
Perceived Care Quality	Relational trust and reassurance	“She knew what she was doing. That made me relax immediately.”	Patient (Both)	6	Patient Satisfaction	Reinforces the importance of observable skill confidence in care evaluation	Social Learning Theory
Outcome Perception Limits	Emergency response delays	“Even if you’re trained, you can’t act fast when there’s no equipment.”	Instructor (Both)	9	Perceived Maternal Complications/Patient Satisfaction	Explains PS → MC non-significance; outcome tied to system, not satisfaction	Organizational Readiness Theory
Outcome Perception Limits	Overload and resource scarcity	“Too many patients, not enough staff. We focus on surviving the shift.”	Staff Nurse (Both)	8	Perceived Maternal Complications	Clinical preparedness is limited by context, not by training alone	Organizational Readiness Theory
Outcome Perception Limits	Patient invisibility of students	“I didn’t even know students were involved until someone told me later.”	Patient (Both)	6	Student Interaction/Patient Satisfaction	Supports SI → PS non-significance; students perceived as irrelevant to care	Situated Learning Theory

Thematic coding was conducted in NVivo 14 following Braun and Clarke’s six-phase framework. Two independent coders iteratively developed and refined code clusters. Saturation was reached by the 13th interview, and inter-coder agreement was high (Cohen’s Kappa = 0.81). An audit trail and reflexive memoing ensured analytic transparency.

These results support Kolb’s Experiential Learning Theory (1984)^59^, which emphasizes active, reflective participation as a foundation for competency development. Students who experienced early clinical exposure and simulation-based practice in the experimental group reported stronger engagement and confidence. However, the absence of a link between student interaction and satisfaction aligns with Informal Learning Theory, highlighting the limitations of passive, unsupervised workplace learning.

The lack of impact from patient satisfaction on perceived maternal complications corroborates findings by a researcher, who cautions against equating perceived care quality with clinical safety. Patients appreciated interpersonal attentiveness, yet institutional delays and resource shortages appeared to override these experiences in affecting outcomes^60^.

The significant path from staff training to satisfaction resonates with Bandura’s Social Learning Theory (1986), which posits that modeled behaviors by skilled staff influence patient perceptions^61^. However, the poor reliability of the staff training construct revealed through weak AVE and CR scores suggests that inconsistent delivery may have undermined its intended impact. This supports Weiner’s Organizational Readiness Theory (2009), which stresses that innovation adoption depends on both cultural and structural institutional preparedness^62^.

### Implications for practice and policy

Several practice and policy insights emerge from the findings. First, the study underscores the importance of implementation fidelity. While the integrated model improved satisfaction, qualitative narratives revealed inconsistent simulation exposure and limited supervisory support. Reform must extend beyond curriculum to include resource provisioning, simulation fidelity benchmarks, and standardized instructional oversight^63^. Second, structured supervision frameworks are essential. Student narratives revealed significant role ambiguity, reducing engagement and learning. Allocating dedicated supervisory hours and embedding real-time task feedback mechanisms could strengthen student-instructor interaction and reinforce clinical readiness. Third, the disconnect between patient satisfaction and clinical outcomes reinforces the need to address system-level deficits. As noted by staff and instructors, institutional gaps, such as understaffing, emergency delays, and inadequate infrastructure, undermined training effectiveness. Alignment with WHO quality of care standards and national vocational training policies is vital to bridge this divide^64^. Lastly, accreditation bodies and oversight agencies should mandate periodic audits focusing not only on curricular content but also on fidelity of simulation environments, supervisory structures, and real-world implementation conditions^65,66^.

This study demonstrates strong methodological alignment with its original proposal. Quantitative instruments showed excellent reliability (α > 0.90), high factorability (KMO = 0.943, Bartlett’s *p* <.001), and strong model performance (R² = 0.743; Q² = 0.717 for patient satisfaction). The use of SmartPLS 4 enabled nuanced exploration of both measurement and structural models, particularly useful in low-variance constructs like perceived maternal complications^67^.

The qualitative strand added valuable context. NVivo 14 facilitated a rigorous thematic analysis of 14 interviews translated from Chinese, ensuring depth and credibility^68^. Inter-coder reliability (Cohen’s Kappa = 0.81) was strong, and theme development was mapped to SEM constructs (see Table 5). The study’s longitudinal structure pre-, mid-, and post-intervention offered stronger causal insights than typical cross-sectional designs in vocational education research. While interviews were professionally translated and cross-checked by a bilingual expert, the possibility of minor conceptual or cultural nuances being lost in translation cannot be entirely ruled out. This represents a common limitation in cross-linguistic qualitative research.

**Table 5:** Multi-Group PLS-SEM results: structural path estimates and measurement metrics for experimental vs. Control Groups.

Measurement & Structural Metrics/Paths	Experimental Group	Control Group	Group Difference Interpretation
Sample Size (n)	60	60	Equal distribution
Staff Training → Patient Satisfaction	β = 0.863, t = 8.123, *p* <.001	β ≈ 0.42, non-significant	Stronger effect in the experimental group
Student Interaction → Patient Satisfaction	β = 0.016, *p* =.720	β ≈ 0.003, non-significant	No significant path difference observed; minimal explanatory contribution in both groups
Patient Satisfaction → Perceived Maternal Complications	β = − 0.124, *p* =.399	β ≈ − 0.07, non-significant	No significant path difference observed; minimal explanatory contribution in both groups
R² – Patient Satisfaction	0.743	< 0.40	High explanatory power only in the experimental group
Q² – Patient Satisfaction	0.717	≈ 0	High predictive relevance in the experimental group
R² – Student Interaction	0.011	0.009	Negligible in both groups
R² – Perceived Maternal Complications	0.015	0.014	Negligible in both groups
CR – Staff Training	0.337	0.361	Poor reliability across both groups (due to low loadings)
CR – Patient Satisfaction	0.932	0.847	Stronger construct reliability in the experimental group
CR – Perceived Maternal Complications	0.931	0.902	Comparable across both groups
CR – Student Interaction	0.900	0.881	Acceptable in both groups
AVE – Patient Satisfaction	> 0.60	~ 0.50	Stronger convergent validity in the experimental group
AVE – Staff Training	0.126	0.138	Very weak in both groups

Table compares path coefficients (β), model explanatory power (R²), predictive relevance (Q²), and reliability/validity metrics (CR, AVE) between students receiving integrated training (experimental) and conventional training (control).

Despite its strengths, the study presents some limitations. The Staff Training construct, while statistically predictive, exhibited poor construct validity (CR = 0.337; AVE = 0.126), requiring refinement of measurement items and construct calibration in future iterations.

It is observed internationally that high-fidelity simulation and structured supervision have the potential to minimize obstetric errors and enhance the work of the team. As an illustration, the UK and Australia simulation programs have reported a decrease in adverse events because of obstetrics when the system level was improved. Nonetheless, implementation studies in LMICs show that perceived competence gains are not always immediately translated into clinical complication reduction because of issues like staffing issues, supply, and poor supervision^18^. This pattern is reflected in our results, which show a significant relationship between staff training and patient satisfaction but no significant decrease in patient-reported complications. This is an indication that although training has the capability of enhancing observable provider behaviors and communication (which patients perceive), systemic preparedness is required to influence clinical safety measures. These results align with Organizational Readiness Theory, which hypothesizes that innovations need the ability and institutional support to alter the outcomes.

Second, this paper primarily focused on post-intervention outcomes. Future research should employ latent growth modeling to examine developmental trajectories over time, particularly concerning student interaction and maternal outcomes. Third, maternal complication data were self-reported rather than extracted from clinical records. Incorporating electronic medical record (EMR) data, such as postpartum hemorrhage rates or neonatal transfer frequency, would improve objectivity and external validity. Finally, the study was conducted within a single institutional framework in China. Broader replication, ideally through multi-site randomized controlled trials with embedded realist evaluation, is necessary to assess generalizability and unpack the contextual mechanisms influencing success.

## Conclusion

This study offers a rigorous, mixed-methods evaluation of an integrated vocational training model designed to enhance maternity care quality in China, a context reflective of challenges faced across low- and middle-income countries (LMICs). Through a quasi-experimental, longitudinal design and thematic integration of stakeholder narratives, the research examined the effects of simulation-based learning, structured supervision, and early clinical immersion on key outcomes^69^. Staff training emerged as a significant predictor of patient satisfaction, consistent with Kolb’s Experiential Learning and Bandura’s Social Learning Theory. Patients valued staff who projected confidence, empathy, and clarity qualities often cultivated through immersive, guided practice. However, the training construct itself showed poor psychometric reliability, a limitation reflected in qualitative accounts of uneven simulation use and inconsistent supervisory protocols. These findings align with Eraut’s Informal Learning Theory, which highlights the risks of unsupervised clinical exposure, and with Weiner’s Organizational Readiness Theory, emphasizing that training outcomes depend as much on institutional capacity as curriculum design. Notably, student interaction showed no significant effect on patient satisfaction, and perceived care quality did not predict perceived maternal complications. Interviews revealed unclear student roles, low engagement, and persistent systemic barriers such as staff shortages and emergency response delays. These limitations underscore the structural constraints impeding the translation of educational reform into measurable clinical outcomes. In summary, while integrated training enhances patient perception and student confidence, its broader clinical impact hinges on implementation fidelity and institutional readiness. To realize its full potential, policymakers and training institutions must go beyond curricular innovation to ensure consistent supervision, high-fidelity simulation environments, and system-level preparedness^70^. This study contributes a scalable evaluation model that bridges educational theory and frontline maternity care, offering transferable lessons for LMICs seeking to strengthen vocational health education.

## Supplementary Information

Below is the link to the electronic supplementary material.

Supplementary Material 1

Supplementary Material 2

## Data Availability

The data used in this study are available as supplementary files S1 and S2.
