# Supplementary material for: An empirical study of integration models and mechanisms for maternity care in innovative vocational education
Source: Sci Rep. 2025 Dec 10;16:2053. doi: 10.1038/s41598-025-31951-w (PMC12808121; doi:10.1038/s41598-025-31951-w)
Supplement: Supplementary file 1 — Supplementary Material 1 [file 41598_2025_31951_MOESM1_ESM.pdf]

Supplementary File S1

Study Questionnaire

Section 1: Demographic Information

Please indicate the most appropriate response for each item below.

1. Age Group
   ☐ Below 20 ☐ 21–25 ☐ 26–30 ☐ 31–35 ☐ Above 35
2. Role in the study:
   ☐ Nursing Student ☐ Staff Nurse ☐ Patient
3. Gender
   ☐ Male ☐ Female ☐ Prefer not to say
4. Highest Education Level
   ☐ Diploma ☐ Bachelor's ☐ Master's ☐ Doctoral ☐ Other: ______
5. Years of Clinical Experience
   ☐ Less than 1 year ☐ 1–3 years ☐ 4–6 years ☐ 7 years and above
6. Role in Facility
   ☐ Student ☐ Staff Nurse ☐ Clinical Instructor ☐ Patient ☐ Other: ______
7. Clinical department/ward:
   ☐ Labour Room ☐ Antenatal Ward ☐ Postnatal Ward ☐ Neonatal Unit ☐ Other:

Section 2: Study Constructs

All items were measured on a 7-point Likert scale:

1 = Strongly Disagree 2 = Disagree 3 = Slightly Disagree 4 = Neutral 5 = Slightly Agree
6 = Agree 7 = Strongly Agree

Construct 1: Staff Training (9 items)

1. ST1 Training sessions improved my confidence in handling maternal care situations.
2. ST2 I received sufficient guidance from supervisors during clinical training.
3. ST3 Simulation-based training enhanced my practical skills.
4. ST4 Trainers demonstrated clear and effective teaching methods.
5. ST5 The training aligned well with real-world clinical practices.
6. ST6 Feedback from instructors helped me improve my performance.
7. ST7 The hospital provided adequate learning resources for training.
8. ST8 Supervision during clinical rotations was consistent and supportive.
9. ST9 Overall, I was satisfied with the quality of staff training provided.

Construct 2: Student Interaction (8 items)

1. SI1 Students communicated effectively with patients during care.
2. SI2 Students collaborated well with the healthcare team.
3. SI3 Clinical instructors encouraged active student participation.

4. SI4 Students demonstrated empathy and professionalism during patient care.
5. SI5 Roles and responsibilities of students were clearly defined.
6. SI6 Feedback between students and mentors was constructive.
7. SI7 Students showed initiative in handling maternal care situations.
8. SI8 Students maintained respectful and ethical behavior with patients.

Construct 3: Patient Satisfaction (7 items)

1. PS1 I felt respected by the healthcare staff during my care.
2. PS2 Staff communicated clearly about my treatment plan.
3. PS3 My privacy was maintained throughout the care process.
4. PS4 I was satisfied with the responsiveness of care providers.
5. PS5 My comfort and safety were prioritized during procedures.
6. PS6 I was encouraged to ask questions about my care.
7. PS7 Overall, I was satisfied with the maternal care I received.

Construct 4: Perceived Maternal Complications (7 items)

1. MC1 I experienced prolonged labor or delivery complications.
2. MC2 I encountered excessive bleeding after delivery.
3. MC3 I developed an infection during or after childbirth.
4. MC4 I experienced delays in receiving care during emergencies.
5. MC5 I felt unsafe or neglected during the childbirth process.
6. MC6 I had difficulty accessing postnatal follow-up services.
7. MC7 My overall recovery after childbirth was affected by complications.
