# Supplementary material for: An empirical study of integration models and mechanisms for maternity care in innovative vocational education
Source: Sci Rep. 2025 Dec 10;16:2053. doi: 10.1038/s41598-025-31951-w (PMC12808121; doi:10.1038/s41598-025-31951-w)
Supplement: Supplementary file 2 — Supplementary Material 2 [file 41598_2025_31951_MOESM2_ESM.pdf]

Supplementary File S2

Semi-Structured Interview Guide

Purpose of the Interview:

To explore participants' experiences with the integrated maternity-care training model, perceptions of its effectiveness, barriers and facilitators to implementation, and how the training influenced clinical practice, communication, and maternal care outcomes.

Interview Format:

Semi-structured, open-ended questions. Interviews last approximately 25–40 minutes.

Interviewers may use follow-up probes to deepen or clarify responses.

Section A. Warm-Up and Background

1. Can you briefly describe your role (student, patient, nurse, instructor) during the period of this training program?
2. Were you directly involved in or exposed to the integrated maternity-care training model?

Section B. Experiences With the Training Model

3. Please describe the training you received during this period.
4. Can you walk me through a specific situation where the training helped you in the clinical setting?
5. If applicable, were there any situations where the training did *not* help or did not match actual clinical needs?

Section C. Supervision and Interaction

6. How would you describe the role of supervisors or clinical instructors during the training? How did the presence of students (or trainees) affect patient care on the ward?
7. For students/instructors/nurses:
8. How would you describe the interaction between students and patients?

Section D. Perceived Outcomes and Patient Care

9. From your perspective, did the training influence the quality of patient care or patient satisfaction?
10. For patients: How satisfied were you with the communication and support you received from staff and students during your care?
11. Did you observe or experience any maternal complications during this period?

Section E. Barriers and Facilitators

12. What challenges or barriers did you notice that limited the effectiveness of the training?
13. What factors helped the training succeed?

Section F. Recommendations

14. If you could improve or change anything about this training model, what would you suggest?
15. Do you think this training model should be expanded to other departments or institutions? Why or why not?
16. Is there anything else you would like to share about your experiences that we have not covered?

Thank the participant for their time and contribution.
